# Simulated responses of permafrost distribution to climate change on the Qinghai–Tibet Plateau

**DOI:** 10.1038/s41598-017-04140-7

**Published:** 2017-06-19

**Authors:** Qing Lu, Dongsheng Zhao, Shaohong Wu

**Affiliations:** 1 Key Laboratory of Land Surface Pattern and Simulation, Institute of Geographic Sciences and Natural Resources Research, Chinese Academy of Sciences, Beijing, 100101 China; 2 University of Chinese Academy of Sciences, Beijing, 100049 China

## Abstract

Climate warming causes changes in permafrost distribution, which affects the surface energy balance, hydrologic cycle and carbon flux in cold regions. In this study, the Surface Frost Number model was applied to examine permafrost distribution on the Qinghai–Tibet Plateau (QTP) under the four RCPs (RCP2.6, RCP4.5, RCP6.0, and RCP8.5). The Kappa statistic was used to evaluate model results by comparing simulations of baseline permafrost distribution (1981–2010) with the existing frozen soil maps. The comparison shows that the Surface Frost Number model is suitable for simulating the general characteristics of permafrost distribution on the QTP. Simulated results suggest that areas of permafrost degradation would be the smallest in the near-term (2011‒2040) with the rates of 17.17%, 18.07%, 12.95% and 15.66% under RCP2.6, RCP4.5, RCP6.0 and RCP8.5, respectively. The rate of permafrost degradation would be faster in the mid-term (2041‒2070), especially under the RCP8.5 scenario (about 41.42%). Areas of permafrost degradation would be the largest in the long-term (2071‒2099) relative to baseline conditions, with a modelled 64.31% decrease in permafrost distribution using the RCP8.5 scenario. Our results would help the decision‒making for engineering construction program on the QTP, and support local units in their efforts to adapt climate change.

## Introduction

Permafrost is defined as ground that remains at or below 0 °C for at least 2 consecutive years^1, 2^. Globally, permafrost occurs across an area of 22.79 × 10^6^ km^2^ and accounts for approximately a quarter of the northern hemisphere land surface area^3^. Permafrost that with an impermeable layer can preserve soil water and protect nutrients for plant growth in arid regions, changes in which have a pronounced effect on regional climate and ecosystems^4^. Permafrost is an important component of the Qinghai–Tibet Plateau (QTP), and its distribution is a key indicator of climatic and environmental change^5^. Permafrost on the QTP is especially sensitive to climate warming^6^. Thawing of the ground can release carbon dioxide to the atmosphere and cause positive feedbacks that contribute to accelerated climate change^7, 8^.

The QTP is more sensitive to climate change and exhibits faster rates of temperature increase than other regions in China. Liu and Chen^9^ used data from 197 observation stations to analyze temperature change on the QTP. They showed that the QTP experienced significant warming since the mid-1950s, especially in winter. The annual rate of temperature increase from 1955 to 1996 was approximately 0.16 °C/decade, and the winter temperature increase was 0.32 °C/decade. This winter increase exceeded other areas at the same latitude in the Northern Hemisphere over the same period^9^. The warming trend was 0.2 °C/decade during the period 1971–2000^10^ and 0.5 °C/decade from 1981–2010^11^. Global temperature is expected to continue to increase^12^: the region along the Qinghai–Tibet railway would warm 2.8–3.0 °C by the middle of the 21^st^ century and 3.8–4.8 °C by the end of the 21^st^ century^13^. Surface soil temperatures in some parts of the QTP have increased of 0.6 °C per decade since 1980^14, 15^, and this warming trend is expected to intensify in the future^13^.

Due to the combined effects of both climate warming and human activity, the QTP is experiencing remarkable permafrost degradation, such as shortened durations of below-freezing temperatures, shrunk permafrost surface areas, deepened the active layer, increased ground temperatures and rose the lower limit of permafrost^14, 16–20^. Permafrost degradation has resulted in a series of environmental issues, including decreases in the number of species, a lower water table, modifications to surface and subsurface hydrology, and changes in energy and carbon exchange between the soil and atmosphere. Loss of permafrost has also threated the safety of engineering construction projects^21–29^. Since extensive permafrost degradation has already caused environmental deterioration, it is of great importance to identify changes in permafrost areas on the QTP to further help identify any ecological or environmental effects.

Because permafrost is a subsurface feature, its distribution cannot be readily estimated using remote sensing data. Further, owning to the high cost and difficulty in accessing many permafrost regions, the distribution is also difficult to map via field investigations. Model simulations are therefore an effective approach, especially for projections of future permafrost distribution. Permafrost distribution on the QTP has been simulated by different models, including the Altitude Model^30^, the Mean Annual Ground Temperature Model^31^, the Response Model^32^ and the Surface Frost Number model^33^. The Surface Frost Number model has been widely used to simulate the change in permafrost distribution all over the world^34–36^. A recent study suggested that global permafrost distribution would obviously retreat northward under RCP4.5, and the permafrost would basically disappear on the Tibetan Plateau^37^. In addition, Nan *et al*. applied a numerical permafrost model of thermal regimes to predict changes in permafrost distribution under two climate warming scenarios^38^. The results indicated that, under a scenario of 0.02 °C/yr air temperature increase, permafrost area on the QTP would decrease by 8.8% in the next 50 years and by 13.4% in the next 100 years. Under a scenario of 0.05 °C/yr air temperature increase, permafrost would decrease by about 13.5% and 46% after 50 and 100 years, respectively. Using the Community Land Model (CLM4), Guo and Wang^19^ simulated near-surface permafrost area as 151.50 × 10^4^ km^2^ on the QTP during the period 1981–2000. While the studies described above all attempted to reveal trends in permafrost distribution, there is uncertainty in the results due to input data and other factors not taken into account in the models.

Previous research on permafrost distribution has often neglected thermal exchange between the atmosphere and land surface through snow, plant and litter cover, which is very important for permafrost occurrence^19, 39^. Moreover, relationships between air and subsurface temperatures are often considered as a linear function to simplify model algorithms that use air temperature or climate scenario data to simulate permafrost distribution. Snow cover is a crucial factor in identifying permafrost^40, 41^, and snow cover duration, density and thickness all exert important impacts on the thermal regimes of soil. Soil moisture controls heat transmission between air and soil^42, 43^, and variation in soil moisture affects temporal and spatial changes in permafrost distribution. Thus, the consideration of the way that thermal regimes of soil moisture, snow, plant and litter cover influence energy transfer between the air and the soil will make the simulation results more reliable and reasonable.

Based on Surface Frost Number model re-drived from an explicit physical formula, Nan *et al*.^35^ found that permafrost distribution is not only decided by surface air temperature, but also obviously affected by soil property, such as soil moisture, soil texture and litter thickness. On the QTP, the plant roots concentrated within the 0~20 cm soil layer^44^, and developed a Mattic Epipedon with the thickness of about 20 cm^45^. In order to decrease the effect of Mattic Epipedon and soil moisture on the soil thermal conduction, the soil temperature at 20 cm depth was used to replace air temperature to calculate the Surface Frost Number model.

In this study, soil temperature at 20 cm depth was simulated by the Lund–Potsdam–Jena (LPJ) dynamic vegetation model that considers the effects of soil moisture, snow depth and litter thickness on soil temperature. This output was used to drive the Surface Frost Number model for simulating permafrost distribution under different climate change scenarios.

## Results

### Simulation of permafrost distribution in the baseline period

The Kappa statistic, which has been widely applied to compare maps with categorical data, was used to evaluate modeling results. Simulated permafrost distribution in the baseline period was compared with the existing 1:4,000,000 Map of the Snow, Ice and Frozen Ground in China^46^ and 1:3,000,000 map of permafrost distribution on the QTP^47^. Monserud and Leemans^48^ proposed that Kappa values of ≥0.8, 0.6–0.8, 0.4–0.6, 0.2–0.4 and 0–0.2 indicate an almost perfect, substantial, high, fair and low fit, respectively, between the two maps.

**Figure 1:**
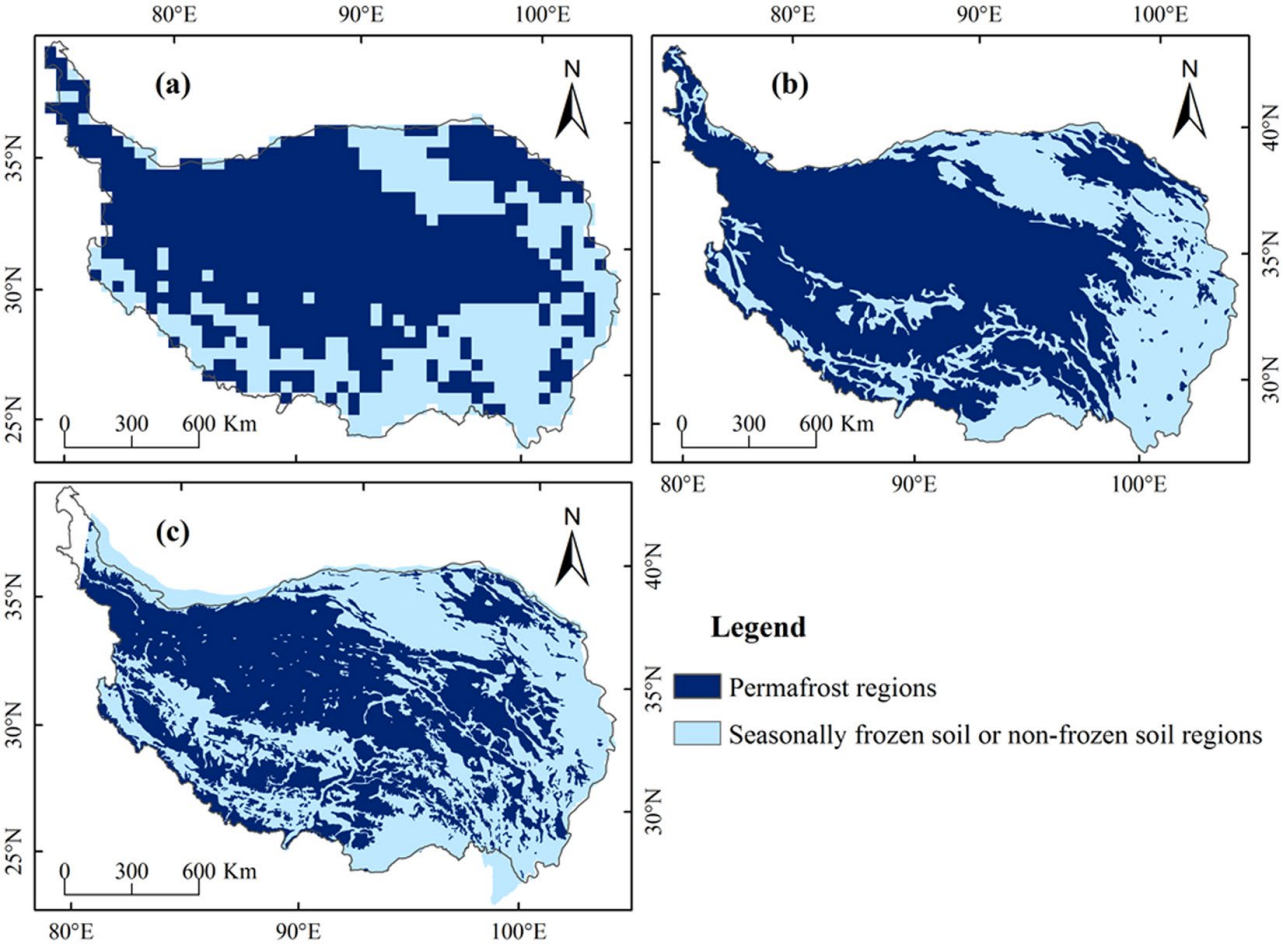
Comparison of simulated permafrost distribution in the baseline period (1981–2010) with the existing frozen soil maps: (**a**) Simulated results in the baseline period (1981–2010), (**b**) frozen soil map from Shi *et al*.^46^ (The data set was obtained from Environmental and Ecological Science Data Center for West China, National Natural Science Foundation of China, http://westdc.westgis.ac.cn), and (**c**) map of permafrost on the QTP from Li and Cheng^47^ (The data set was obtained from Cold and Arid Regions Sciences Data Center at Lanzhou, http://westdc.westgis.ac.cn). (The figure was created using ArcGIS 10.1 http://www.esri.com/ software).

Figure 1a shows simulated permafrost distribution on the QTP in the baseline period, derived from mean F_+_ values during the period 1981–2010. The area of permafrost accounts for about 64.91% (about 1.66 × 10^6^ km^2^) of the QTP in the baseline period, while permafrost area is 1.61 × 10^6^ km^2^ from Shi *et al*.^46^ and 1.36 × 10^6^ km^2^ from Li and Cheng^47^. Among of them, the area of seasonally frozen soil or non‒frozen soil are 0.90 × 10^6^ km^2^, 0.96 × 10^6^ km^2^, and 1.26 × 10^6^ km^2^ for the results from simulation, Shi *et al*.^46^ and Li and Cheng^47^, respectively.

A comparison of simulated results with frozen ground types from Shi *et al*.^46^ (Fig. 1b) and Li and Cheng^47^ (Fig. 1c) shows that the spatial distribution of modeled permafrost distribution is consistent with the Kappa value of 0.51 and 0.46, respectively. The result is considered a high agreement according to criteria from Monserud and Leemans^48^. In addition, we calculated the proportion of the same frozen ground type area between modeled result in the baseline period and existing frozen soil map from Shi *et al*.^46^ and Li and Cheng^47^, and the consistencies were 75.4% and 70.2%, respectively. Simulated permafrost distribution in the eastern QTP is slightly larger than for the two frozen ground maps, with seasonal frozen ground displaced by permafrost in the northern Gangdise and Himalaya mountains. Compared with the frozen ground maps, simulated permafrost distribution in the southern Qiangtang Plateau is significantly larger than that from Li and Cheng^47^, and similar to the result from Shi *et al*.^46^. This study classifies predominantly continuous permafrost, isolated permafrost and alpine permafrost as permafrost, and the other areas as seasonally frozen ground. In the simulation results, permafrost is mainly distributed in regions between the Kunlun and Gangdise mountains, and the Qilian mountains.

### Changes in permafrost distribution under RCP scenarios

**Figure 2:**
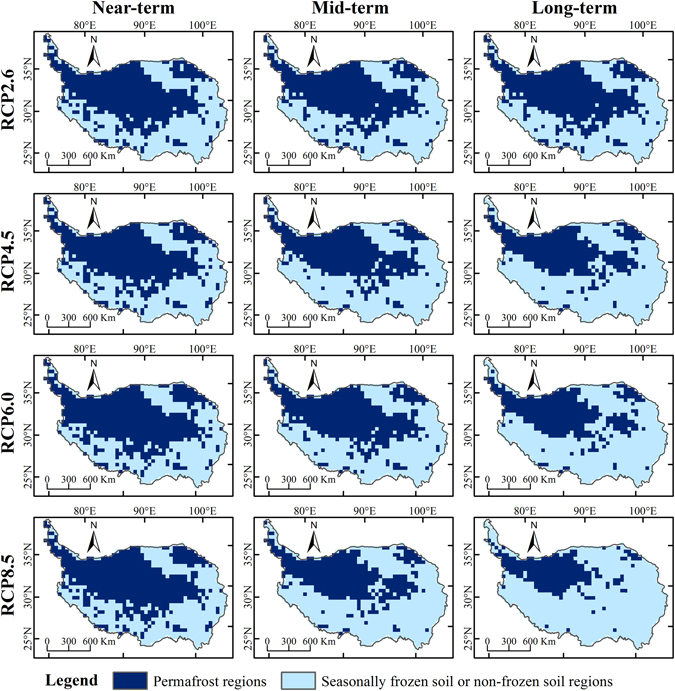
Permafrost distribution on the QTP in the near-term (2011–2040), mid-term (2041–2070) and long-term (2071–2099) under RCP2.6, RCP4.5, RCP6.0 and RCP8.5 scenarios. (The figure was created using ArcGIS 10.1 http://www.esri.com/ software).

The distribution of permafrost can be affected by climate change. Permafrost distribution in the near-term, mid-term and long-term under different climate change scenarios is illustrated in Fig. 2. The spatial distribution of permafrost is similar in the three periods under the RCP2.6 scenario, implying that permafrost would not be greatly affected by climate change. Changes in permafrost under RCP4.5 and RCP6.0 scenarios show a similar pattern, with the permafrost area being largest in the near-term, followed by the mid-term and then long-term. The degradation of permafrost under the RCP8.5 scenario is more severe than for other scenarios, especially in the long-term.

**Figure 3:**
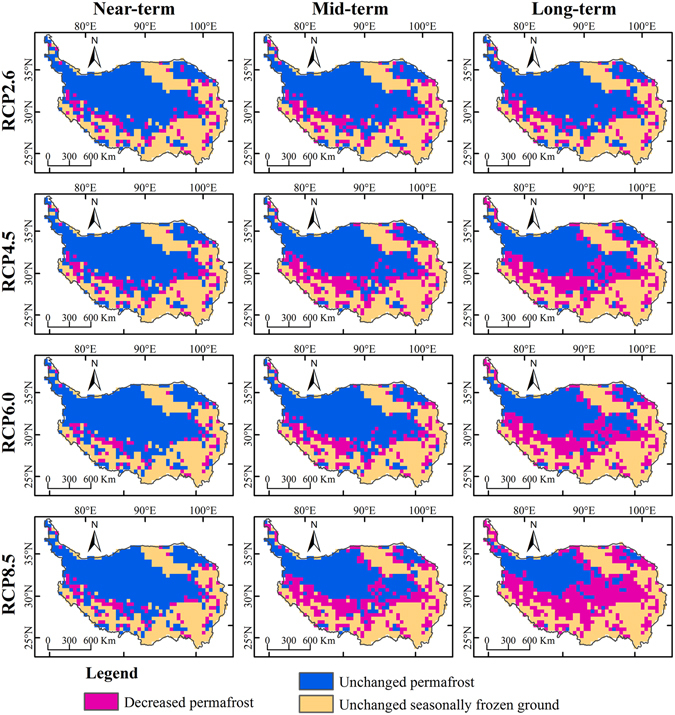
Spatial distribution of changes in permafrost area on the QTP in the near-term (2011–2040), mid-term (2041–2070), long-term (2071–2099) compared with the baseline period under RCP2.6, RCP4.5, RCP6.0 and RCP8.5 scenarios. Decreased permafrost area indicates that permafrost has degraded to seasonally frozen or non-frozen regions; unchanged permafrost is permafrost that has persisted over different time periods; unchanged seasonally frozen ground indicates areas that are persistently non-permafrost regions. (The figure was created using ArcGIS 10.1 http://www.esri.com/ software).

Figure 3 and Table 1 show changes in permafrost distribution on the QTP during different periods under RCP2.6, RCP4.5, RCP6.0 and RCP8.5 scenarios compared with the baseline period. Under the four climate change scenarios, a slight degradation in permafrost is observed in the near-term. Areas of decreased permafrost are distributed at the boundary between permafrost and seasonally frozen ground, and are mainly located in the southwestern and eastern QTP. The RCP4.5 scenario shows the most severe degradation of permafrost in the near-term, with a permafrost decrease of 0.30 × 10^6^ km^2^, accounting for 18.07% of total permafrost in the baseline period. The lowest permafrost degradation in the near-term occurs under the RCP6.0 scenario, with a decrease of 0.22 × 10^6^ km^2^. This is followed by the RCP8.5 scenario with a decrease of 0.26 × 10^6^ km^2^ and the RCP2.6 scenario with a decrease of 0.29 × 10^6^ km^2^.

**Table 1:** Area (10^6^ km^2^) and percentage (%) of decreased permafrost on the QTP in the near-term, mid-term and long-term compared with the baseline period under RCP2.6, RCP4.5, RCP6.0 and RCP8.5 scenarios.

Different periods	RCP2.6		RCP4.5		RCP6.0		RCP8.5	
	Decreased area (10^6^ km^2^)	Decreased percentage (%)	Decreased area (10^6^ km^2^)	Decreased percentage (%)	Decreased area (10^6^ km^2^)	Decreased percentage (%)	Decreased area (10^6^ km^2^)	Decreased percentage (%)
Near-term	0.29	17.17	0.30	18.07	0.22	12.95	0.26	15.66
Mid-term	0.40	23.95	0.54	32.53	0.45	26.96	0.69	41.42
Long-term	0.37	22.44	0.73	43.67	0.77	46.08	1.07	64.31

In the mid-term, areas of decreased permafrost are mainly distributed in the southern Qiangtang Plateau, the eastern QTP and the surrounding Qilian mountains. In contrast to near-term simulations, the RCP8.5 scenario exhibits the largest decrease in permafrost area, while the RCP2.6 scenario shows the smallest decrease. Compared with the baseline period, the decrease in permafrost area in the mid-term is fastest under the RCP8.5 scenario, with a decrease in area of about 0.69 × 10^6^ km^2^. This accounts for 41.42% of total permafrost area in the baseline period. A slight degradation is found using the RCP2.6 scenario, with a permafrost decrease of 0.11 × 10^6^ km^2^ compared with the near-term. The decrease in permafrost area is 0.54 × 10^6^ km^2^ under the RCP4.5 scenario and 0.45 × 10^6^ km^2^ under the RCP6.0 scenario in the mid-term relative to the baseline period. The decrease in area in the mid-term under the RCP4.5 and RCP6.0 scenarios is similar to the near-term with losses of 0.24 × 10^6^ km^2^ and 0.23 × 10^6^ km^2^, respectively.

In the long-term, the most severe degradation of permafrost occurs under the RCP8.5 scenario, with a decrease in permafrost area of about 1.07 × 10^6^ km^2^, which represents a loss of up to 64.31%. Compared with the mid-term, the loss of permafrost area is about 0.38 × 10^6^ km^2^, which is equal to permafrost degradation in the mid-term relative to the near-term. Areas of decreased permafrost are mainly located in the southern Qiangtang Plateau, eastern QTP and Qilian mountains. The area of permafrost decrease under the RCP6.0 scenario is 0.77 × 10^6^ km^2^, and residual permafrost is concentrated in the central QTP. Under the RCP4.5 scenario, the rate of decrease in permafrost area is slower than under RCP6.0 and RCP8.5 scenarios, with a decreased area of 0.19 × 10^6^ km^2^ in long-term relative to mid-term. In contrast to other scenarios, the RCP2.6 simulation shows areas of increased permafrost in the Gangdise mountains.

## Discussion

**Figure 4:**
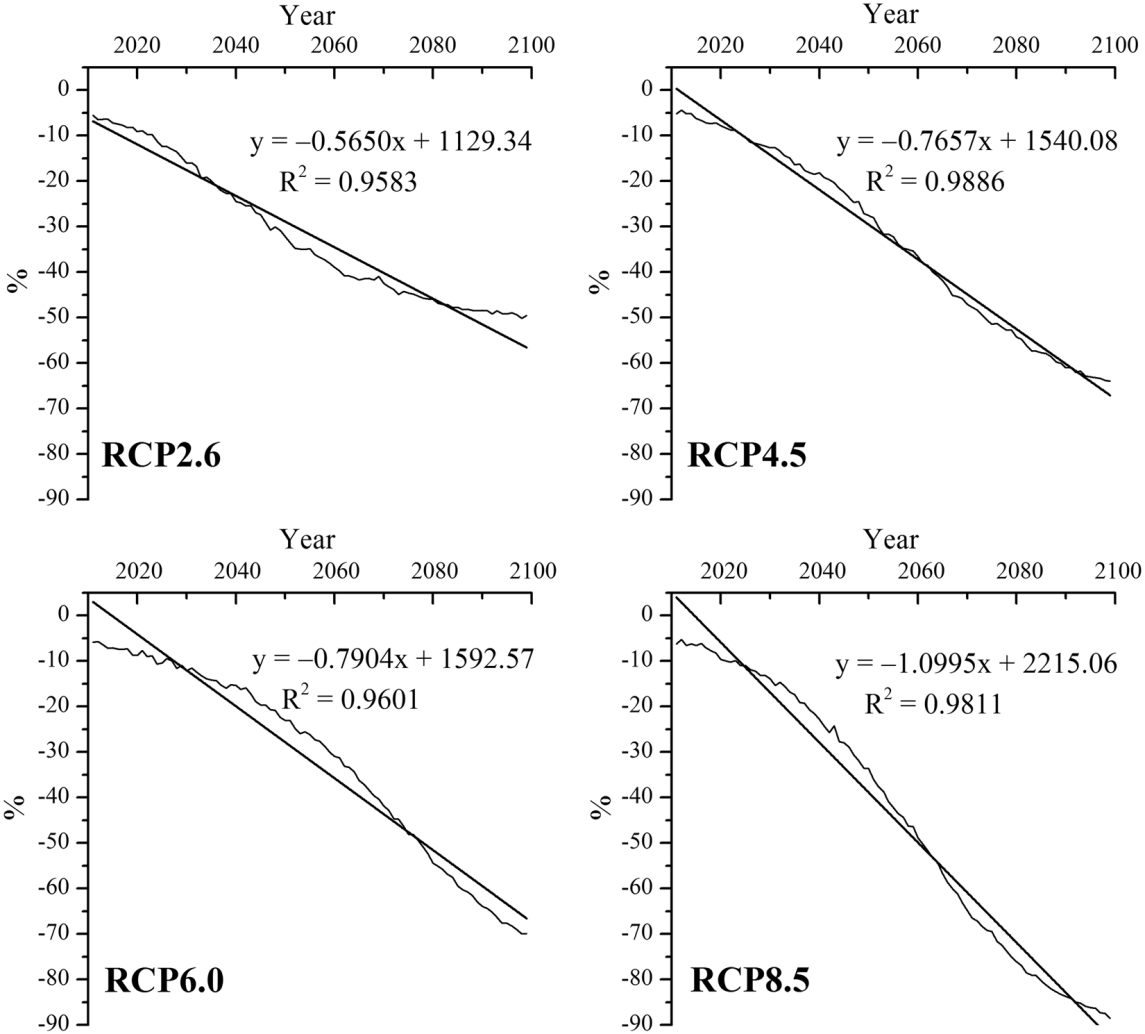
Inter-annual percentage variations in snow depth on the QTP relative to the average of the baseline term (1981‒2010) under RCP2.6, RCP4.5, RCP6.0 and RCP8.5 scenarios.

In this research, we used the Surface Frost Number Model to simulate permafrost and seasonal frozen soil or non-frozen soil distribution on the QTP over different time periods. Snow is critical for atmosphere and lithosphere coupling^49, 50^, and snow cover has been shown to be a key factor in controlling ground temperatures and determining the development of permafrost^50^. Jin *et al*.^51^ indicated ground temperature in permafrost regions of the QTP not only are controlled by regional zonations of latitude, longitude and elevation, but also significantly affected by local environmental factors including vegetation, snow cover, surface water conditions, and geological structure. The distribution of snow depth on the QTP shows obviously spatial heterogeneity, which snow cover in mountainous regions of the eastern, southern, and interior QTP are deeper than those in high plains, valleys and basins in the interior of QTP. The snow cover is thick and has long duration in the area with stability of snow, which plays a role in insulating thermal for the shallow ground. If snow thick is more than 20 cm, the role of insulation will be enhanced. However, in the area with thinner snow in the warm season, the influence of snow cover on the shallow ground will be cooling due to the existence of snow with short time^51^. Therefore, snow cover on the QTP might heavily affect the thermal regime of permafrost. Inter-annual percentage variations in snow depth on the QTP relative to the average of the baseline term under the four RCP scenarios are shown in Fig. 4. The annual snow depth of the four RCP scenarios show a significant decrease trend (*P* < 0.001), especially under the RCP8.5 scenario. The annual snow depth under RCP8.5 scenario would decrease at a rate of approximately 12.49 mm/yr from 2011 to 2099. Moreover, soil moisture also controls heat transmission between air and soil. Therefore, permafrost distribution assessments based on energy balance^52^ or statistical modeling^53^ may overestimate permafrost area due to neglecting the influence of snow, litter cover and soil moisture. These uncertainties are particularly relevant to simulations of future permafrost distribution. This study therefore uses the Surface Frozen Number model to include the influences of heterogeneous snow cover and soil moisture on the ground thermal regime under different RCP scenarios.

The Kappa statistic was used to measure agreement between the simulated and the observed frozen soil maps. The Kappa value of over 0.5, suggested that simulation in the baseline period agreed‒well with the frozen soil maps from Shi *et al*.^46^ and Li and Cheng^47^. The area of simulated permafrost of 1.66 × 10^6^ km^2^ in the baseline period was larger than those from Shi *et al*.^46^ and Li and Cheng^47^. Compared with the simulated permafrost area with result from Shi *et al*.^46^, differences primarily concentrated in the eastern QTP and the northern Gangdise and Himalaya mountains. There were differences in spatial distribution of permafrost between Li and Cheng^47^ and simulation in the baseline period, which could contribute to the discrepancy of permafrost area. Figure 1c showed that the regions different from simulation were covered by seasonally frozen soil ground or non‒frozen soil ground. Therefore, the area of seasonally frozen soil ground or non‒frozen soil ground in Li and Cheng^47^ was significantly larger than those in the simulation. Moreover, these inconsistencies could be partly attributed to the relatively large grid size of the model and the climate data used as input in the baseline period. Climate data in the baseline period was interpolated to a resolution of 0.5° × 0.5° using a thin-plate smoothing spline algorithm with meteorological station data and latitude, longitude and elevation as independent variables. However, meteorological stations in the QTP are unevenly distributed with few observations in the northwestern QTP, which is a large permafrost region. Moreover, there are few representative observation stations in the eastern QTP because of its complex topography. The uneven distribution of meteorological stations may have affected the accuracy of the simulated results.

The total area of simulated permafrost in the baseline period on the QTP is 1.66 × 10^6^ km^2^ (including glaciers, lakes and rivers) which is larger than simulation (1.27 × 10^6^ km^2^) from Chang *et al*.^36^ using the Surface Frozen Index model during the period 1986‒2005. Although the Surface Frozen Index model was used in the two studies, some differences still contributed to the area of inconsistency. Firstly, F_+_ value of 0.58 was used by Chang *et al*.^36^ to distinguish permafrost regions and seasonal frozen ground regions in order to match permafrost area with result from Li and Cheng^47^, while 0.50 was used in this study. Based on Surface Frost Number model re‒drived from an explicit physical formula, Nan *et al*.^35^ suggested that F value of 0.50 is more reasonable for distinguishing permafrost regions and seasonal frozen ground regions which should consider the effect of soil property on the freezing and thawing process. Secondly, different spatial resolution of permafrost distribution may contribute to the discrepancies. Moreover, the permafrost area in our study is also larger than the results from Guo *et al*.^19^ using the Community Land Model also over the period from 1981 to 2010. Differences may relate to the QTP boundaries, scale of the simulations and whether the simulation excludes glaciers and lakes. Further, Guo and Wang^19^ used air temperature as input data and neglected the thermal conduction of snow cover. Studies by Li and Cheng^54^ and Zhang *et al*.^55^ resulted in areas of permafrost were 1.294 × 10^6^ km^2^ and 1.48 × 10^6^ km^2^, respectively. In both cases, an altitude model was used to predict permafrost distribution which only uses elevation as input data and this does not reflect the response of permafrost area to changes in temperature, snow, precipitation, solar radiation, and especially, the influence of snow cover. An altitude model cannot be used to predict future permafrost distribution. Furthermore, the resolution of the digital elevation model (DEM) was 30-second (latitude and longitude) in Li and Cheng^54^ and 1 km in Zhang *et al*.^55^, which would also contribute to discrepancies with the results of the present study. Guo and Wang^19^ indicated that a higher-resolution model might improve the simulation accuracy. As the objective of the present study was to produce projections of permafrost distribution and analyze the rate of change under different climate change scenarios, we used input data at a resolution of 0.5° × 0.5°. However, methods using higher-resolution climate data should be developed in the future to further improve simulation accuracies.

Differences between the permafrost map and simulation results may be attributed to several factors. Firstly, there are differences in map boundaries and scale, and the period of data used to produce the map compared with the model. Secondly, applied models are vastly different and also use a wide range of input datasets at various resolutions. Additionally, only a select number of models consider the influence of the thermal regimes of snow, plant and litter cover on energy transfer between the air and the soil, and the effect of land-types, lakes, glaciers and deserts that may all change in response to climate.

Under climate warming scenarios, the permafrost distribution on the QTP is expected to shrink toward the northwest. In the long-term, permafrost areas on the QTP are predicted to decrease by 22.44%, 43.67%, 46.08% and 64.31% under RCP2.6, RCP4.5, RCP6.0 and RCP8.5 scenarios, respectively. The rate of decrease under the RCP8.5 scenario is similar to results from Zhang *et al*.^55^, who used an altitude model and temperature lapse rate (VLRT) data to suggest a loss in permafrost area of 62.84% by 2070. The altitude model used by Zhang *et al*.^55^ neglected permafrost distribution responses to climate change, and climate data from 1950 to 2000 were used as a baseline for estimating changes in permafrost area in the future. In the present study, climate data from 1981–2010 were used for the baseline period, which is more reliable for simulation accuracy assessment.

Based on a numerical permafrost model, Nan *et al*.^38^ indicated that significant degradation would occur after 100 years, with a loss in area of about 46% with a rate of temperature increase of 0.052 °C/yr. Li and Cheng^54^ also used an altitude model to show that loss of permafrost area would exceed 58% if air temperature increased on the QTP by an average of 2.91 °C by 2099. Reasons for discrepancies between these studies and the present analysis may be differences in temperature increase scenarios and the lack of consideration of snow cover in previous research.

Temperature is a fundamental factor in controlling permafrost distribution. Global mean surface temperature is projected to increase 1.5 °C under the lowest-emission RCP scenario and 4.5 °C under the highest-emission RCP scenario relative to pre-industrial levels by 2100^56^. In China, the projected rate of temperature increase would be 0.06 °C/decade and 0.63 °C/decade under RCP2.6 and RCP8.5 scenarios, respectively, and warming trends would be stronger in the northern regions than in the southern regions^57^. The decrease in permafrost area would be more pronounced under the RCP8.5 scenario (approximately 62.0%) than under the RCP2.6 scenario (approximately 18.7%), which would be consistent with climate warming until the end of the 21^st^ century. A significant spatial correlation was found between the remaining permafrost distribution^57^ and the extent of temperature increase under the condition of permafrost existing. Under the RCP2.6 scenario, temperature would increase slightly in all time periods, corresponding to slight changes in permafrost area. In this projection, sporadic and discontinuous permafrost would disappear in the mid-term in the eastern QTP and Gangdise mountains; by the end of the 21^st^ century, the greatest temperature increase would occur in the Gangdise mountains and the southern Qiangtang Plateau, and continuous permafrost in the southern Qingtang Plateau would shrink northward.

Under all climate change scenarios, permafrost is projected to only exist in the northwestern and northeastern QTP, and almost all permafrost in the southern and eastern QTP would disappear. These results are similar to those of Nan *et al*.^38^ and Li and Cheng^54^, which show that permafrost is expected to only persist in the Qiangtang continuous permafrost zone, and in the Gangdise and Qilian mountains. In the present study, however, there were projected disappearance of permafrost distribution in the Gangdise mountains, and this may be associated with the climate forcing data from the RCP scenarios. Differences may also relate to the fact that snow cover and soil moisture were taken into account in estimating the ground thermal regime in our study. Permafrost area in the eastern and southern QTP and in the foothills surrounding high-elevation areas facing the plateau are expected to decrease more rapidly than in other regions. In general, a permafrost degradation is expected to increase from the outer to the inner plateau.

## Conclusions

Using the Surface Frozen Number Model, this study adjusts the “surface frozen number” F_+_ value to classify frozen soil areas with F_+_  ≥ 0.5 as regions of permafrost and other areas as seasonally frozen soil or non-frozen soil ground. Simulations of permafrost distribution were produced for the baseline period, near-term, mid-term, and long-term under RCP2.6, RCP4.5, RCP6.0 and RCP8.5 scenarios. Rates of permafrost degradation were also analyzed between different periods under different scenarios.

The area of simulated permafrost in the baseline period is 1.66 × 10^6^ km^2^. Permafrost area on the QTP is expected to decrease in response to climate warming. In the near-term, permafrost degradation is projected to result in an average loss of about 18% for all climate change scenarios. The rate of permafrost decrease is expected to be faster in the mid-term with the largest degradation rate under RCP8.5 and the smallest under RCP2.6. In the long-term, permafrost areas on the QTP are projected to decrease by 22.44%, 43.67%, 46.08% and 64.31% under RCP2.6, RCP4.5, RCP6.0 and RCP8.5 scenarios, respectively. Permafrost degradation would mainly occur in the eastern and southern QTP and around the boundaries of the continuous permafrost zone. By the end of the 21^st^ century, simulations show that most permafrost would have retreated into the Qiangtang Plateau.

## Materials and Methods

### Study area

The QTP is located in south-central Asia and southwestern China, mainly in Qinghai Province and the Tibet Autonomous Region. It extends from 73.4°E to 104.5°E and from 25.8°N to 104.5°N, and has a total area of 2.65 × 10^6^ km^2^ ^58^. As a result of an average elevation of more than 4000 m above sea level^9^, the QTP has long been known as the “roof of the world” or “the third pole”.

**Figure 5:**
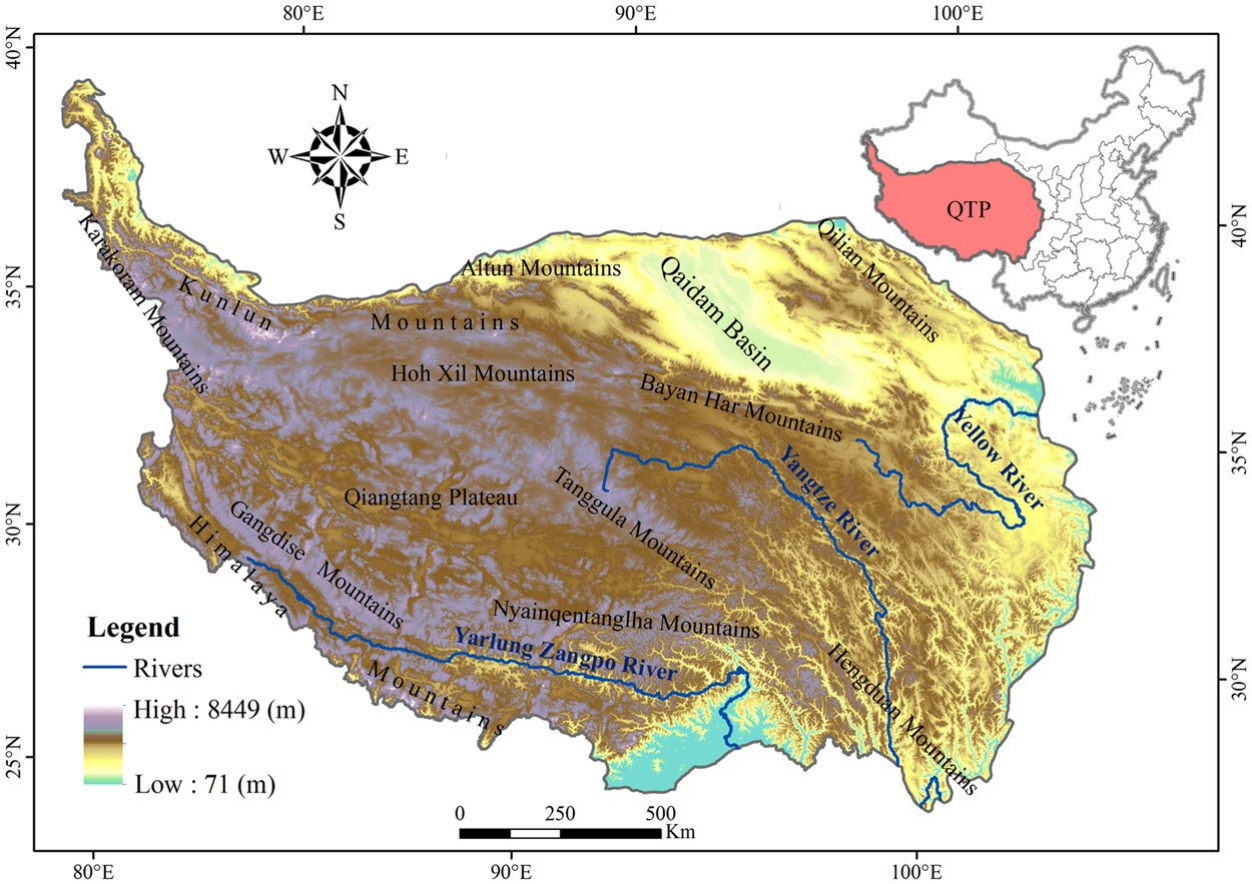
The QTP showing main rivers and geomorphologic units. (The figure was created using ArcGIS 10.1 http://www.esri.com/ software).

The QTP comprises a series of high mountain ranges including the Himalaya, Kunlun, Qilian, Gangdise, Tanggula and Hengduan mountains. It also encompasses two relatively flat regions, Qiangtang Plateau and Qaidam Basin. The headwaters of the Yangtze, Yellow and Yarlung Zangpo rivers all originate in the QTP (Fig. 5), and the region is also characterized by large areas of glaciers, alpine lakes and swamps. Permafrost on the QTP has the highest and largest area of all low and middle latitude regions on earth, and covers more than half of the total area of the QTP^59^. Because of its high altitude and complex topography, the QTP exerts a strong influence on both regional and global climate and produces a distinctive atmospheric circulation pattern^60^.

The QTP acts as a barrier that effectively blocks cold air moving south into South Asia. The Himalaya mountains also prevent the warm and wet airflow from moving north. Therefore, the QTP is affected by both southeast and southward moving monsoons that transport dry winds from Central Asia across the region.

### Methods

Permafrost distribution was simulated using the Surface Frost Number model, developed from the Air Frost Number model of Nelson and Outcalt^33^. To make simulation results more reliable, air temperature in the Air Frost Number model was replaced by soil temperature at 20 cm depth in the LPJ model to consider the effects of snow cover, soil moisture and other variables on temperature conductivity. All soil temperatures used in the analysis are for a depth of 20 cm and all temperatures are expressed in degrees Celcius. The Surface Frost Number *F*_+_, where the subscript (+) represents an adjustment of air temperatures for the effects of snow cover, is defined as:

1 $${F}_{+}=\frac{\sqrt{{DD}{F}_{+}}}{\sqrt{{DD}{F}_{+}}+\sqrt{DDT}}$$

where *DDF* and *DDT* are freezing and thawing indices (degree days) that denote seasonal degree-day sums above and below 0 °C, respectively. Since these parameters are often not readily available, changing surface temperature curves are taken to approximate cosine functions. These functions are used to acquire the following freezing and thawing indices:

21 $$\bar{{T}}=({\bar{{T}}}_{{h}}+{\bar{{T}}}_{{c}})/2$$

22 $$A=({\bar{T}}_{h}-{\bar{T}}_{c})/2$$

23 $$\beta ={\cos }^{-1}(-\bar{{T}}/{A})$$

24 $${\bar{{T}}}_{{s}}=\bar{{T}}+{A}(\sin \,\beta /\beta )$$

25 $${\bar{{T}}}_{{w}}=\bar{{T}}-{\rm{A}}[\sin \,\beta /(\pi -\beta )]$$

26 $${L}_{s}={\rm{365}}(\beta /\pi )$$

27 $${L}_{w}=365-{L}_{s}$$

28 $${DDT}={\bar{{T}}}_{s}\cdot {L}_{s}$$

29 $${DD}{F}_{+}=-{\bar{{T}}}_{w}\cdot {L}_{w}$$

where $\bar{{T}}$ is the mean annual soil temperature, *A* represents the annual soil temperature amplitude, ${\bar{{T}}}_{{h}}$ and ${\bar{{T}}}_{{c}}$ are mean annual soil temperatures in the warmest and coldest months, *β* is the “frost angle” or point on the time axis where the soil temperature curve crosses 0 °C and ${\bar{{T}}}_{{s}}$ and ${\bar{{T}}}_{{w}}$ are the mean summer and winter soil temperature.

A F_+_ value of 0.5 was used in this study, which is considered a reasonable threshold to distinguish between permafrost regions and seasonal frozen ground regions^35, 61, 62^. Raster values of ≥0.5 and <0.5 are taken as a permafrost regions and seasonal frozen soil or non-frozen soil regions, respectively.

In this study, the LPJ model^63, 64^ was adopted to simulate soil temperatures at 20 cm depth under various climate change scenarios on the QTP. LPJ is an intermediate complex dynamic vegetation model that was developed based on an early equilibrium model (BIOME3), and incorporates many features of BIOME series models. LPJ considers ecosystem processes and land-atmosphere carbon and water exchanges that were combined in a modular framework. The LPJ model uses the semi-empirical approach of Haxeltine and Prentice^65^ to model soil hydrology including snowmelt, freeze/thaw, percolation, rainfall, evapotranspiration and runoff. A description of soil hydrology in the model is given by Gerten *et al*.^64^. In the LPJ model, soil temperature follows surface ground temperature with a dampened oscillation. This oscillation is a function of surface ground temperature and soil thermal diffusivity, which depends on soil texture and soil water content. Soil temperature at depth *z* and time *t* are assumed to comply with an annual sinusoidal cycle of air temperature^66^. The formula is as follows:

3 $$T(z,T)={T}_{ave}+A\cdot \exp (-z\cdot {d}^{-1})\sin ({\rm{\Omega }}\cdot {\rm{\Delta }}t-z\cdot {d}^{-1})$$

Here, *T*_ave_ represents the mean annual temperature above a respective layer and *A* represents the amplitude of the temperature fluctuation. ${\rm{d}}=\sqrt{2{k}\cdot {{\Omega }}^{-1}}$ is the damping depth with thermal diffusivity ${k}={\rm{\lambda }}\cdot {{c}}^{-1}$, where *c* is volumetric heat capacity and *λ* is thermal conductivity. *Ω* is the angular frequency of oscillation.

The soil temperature is the result of air temperature influenced by snow, litter and soil water content. The calculation processed are expressed as follows:

4 $${{T}}_{{ave}}={{T}}_{{a}}+{\rm{\Delta }}{{T}}_{{sn}}+{\rm{\Delta }}{{T}}_{{lit}}$$

5 $${A}={{A}}_{{a}}-{\rm{\Delta }}{{A}}_{{sn}}-{\rm{\Delta }}{{A}}_{{lit}}$$

6 $${k}=\{\begin{array}{l}\frac{{k}_{2}-{k}_{1}}{{\rm{0.15}}}\times w+{k}_{1},\quad \quad w < {\rm{0.15}}\\ \frac{{k}_{3}-{k}_{2}}{{\rm{0.85}}}\times (w-{\rm{0.15}})+{k}_{2},w\ge {\rm{0.15}}\end{array}$$

where *T*_*a*_ is annual mean air temperature (°C); *A*_*a*_ is amplitude of annual mean air temperature fluctuation; *ΔT*_*sn*_, *ΔT*_*lit*_, *ΔA*_*sn*_, *ΔA*_*lit*_ (subscript *sn* is the snow, and *lit* is the litter) represent the effects of snow and litter on temperature conduction, respectively; *k*_*1*_ is thermal diffusivity at wilting point (mm^2^/s) (water holding capacity is 0%), *k*_*2*_ is thermal diffusivity at water holding capacity of 15%(mm^2^/s), *k*_*3*_ is thermal diffusivity at field capacity (mm^2^/s) (water holding capacity is 100%); *w* is soil water content.

In LPJ model, the soil temperature is a function of surface ground temperature and soil thermal diffusivity. In equation 3, volumetric heat capacity (*c*) and thermal conductivity (λ) are not constants, which are derived from mean volumetric water content. The nonlinear relationship λ = λ(*w*) is approximated linearly for *w* ∈ [0.1…0.25) and *w* ∈ [0.25…1), respectively. While a phase change the latent heat Q is considered as apparent heat capacity^67^. LPJ considered effects of soil moisture, snow depth, Mattic Epipedon, and litter thickness on the soil thermal properties. Therefore, we used the soil temperature at 20 cm depth which modeled by LPJ model to drive the Surface Frozen Number model.

The LPJ model was modified for use in China using plant function types and an evaporation algorithm^68, 69^. Compared with observed data, the modified LPJ model was shown to effectively simulate variations in net primary productivity, soil carbon, soil moisture and actual evapotranspiration^11, 70, 71^.

### Data

Climate data from 603 meteorological stations in China for the period 1981 to 2010 were obtained from the National Meteorological Center of the China Meteorological Administration (CMA). Data were interpolated with a spatial resolution of 0.5° × 0.5° using the thin-spline plate method. Climate variables, including daily mean, maximum and minimum air temperatures, mean relative humidity, mean wind speed and sunshine duration were extracted from the dataset for the QTP. These data were then used to force simulated permafrost distribution. Data gaps were filled using long-term average values from the same station.

Forcing data were acquired from five GCMs participating in the Coupled Model Intercomparison Project Phase 5 (CMIP5) experiment^72^, including HadGEM2-ES^73^, IPSL-CM5A-LR^74^, GFDL-ESM2M^75^, MIROC-ESMCHEM^76^ and NorESM1-M^77^. GCM output covers the period 1981–2099, and was downscaled to a spatial resolution of 0.5° × 0.5° by the Inter-Sectoral Impact Model Intercomparison Project (ISI-MIP). A bias correction was performed on the ISI-MIP dataset using a statistical approach to preserve absolute or relative trends in simulated daily climate data, with the bias‒corrected daily variables including the average, maximum, and minimum temperatures (K); precipitation (kg·m^−2^·s^−1^); shortwave and longwave downwelling radiation (W·m^−1^); near‒surface wind speed (m·s^−1^); and relative humidity (%)^78^. The ensemble average was employed in this study to reduce uncertainty in GCM performance. Moreover, anomalies calculated from the difference between observed data and ISI-MIP data over the baseline period (1981–2010) were added to scenario data to provide model input for the scenario period (2011–2099).

To estimate future permafrost distribution, the GCM ensemble average for each F_+_ value was calculated for each of the four emission scenarios: RCP2.6, RCP4.5, RCP6.0 and RCP8.5. Four 30-year periods were used to analyze spatial and temporal variations in permafrost distribution as follows: the baseline period (1981–2010); and near-term (2011–2040), mid-term (2041–2070) and long-term (2071–2099) periods. Rates of permafrost change were calculated for each grid as the differences between the baseline period and the near-term, mid-term and long-term periods under different scenarios. Albers equal-area projection was applied during the computation to eliminate area distortion.

The 1:4,000,000 Map of the Snow, Ice and Frozen Ground in China^46^, provided by Environmental and Ecological Science Data Center for West China, National Natural Science Foundation of China (http://westdc.westgis.ac.cn), and 1:3,000,000 Map of permafrost distribution on the QTP^47^, provided by Cold and Arid Regions Sciences Data Center at Lanzhou (http://westdc.westgis.ac.cn) were used to analyze the differences between them and simulated results during the baseline period (1981–2010). The existing frozen soil maps were produced using observational borehole, terrain and remote sensing data, and are currently thought to be a reasonable approximation of frozen soil distribution on the QTP^32^.

**Figure 6:**
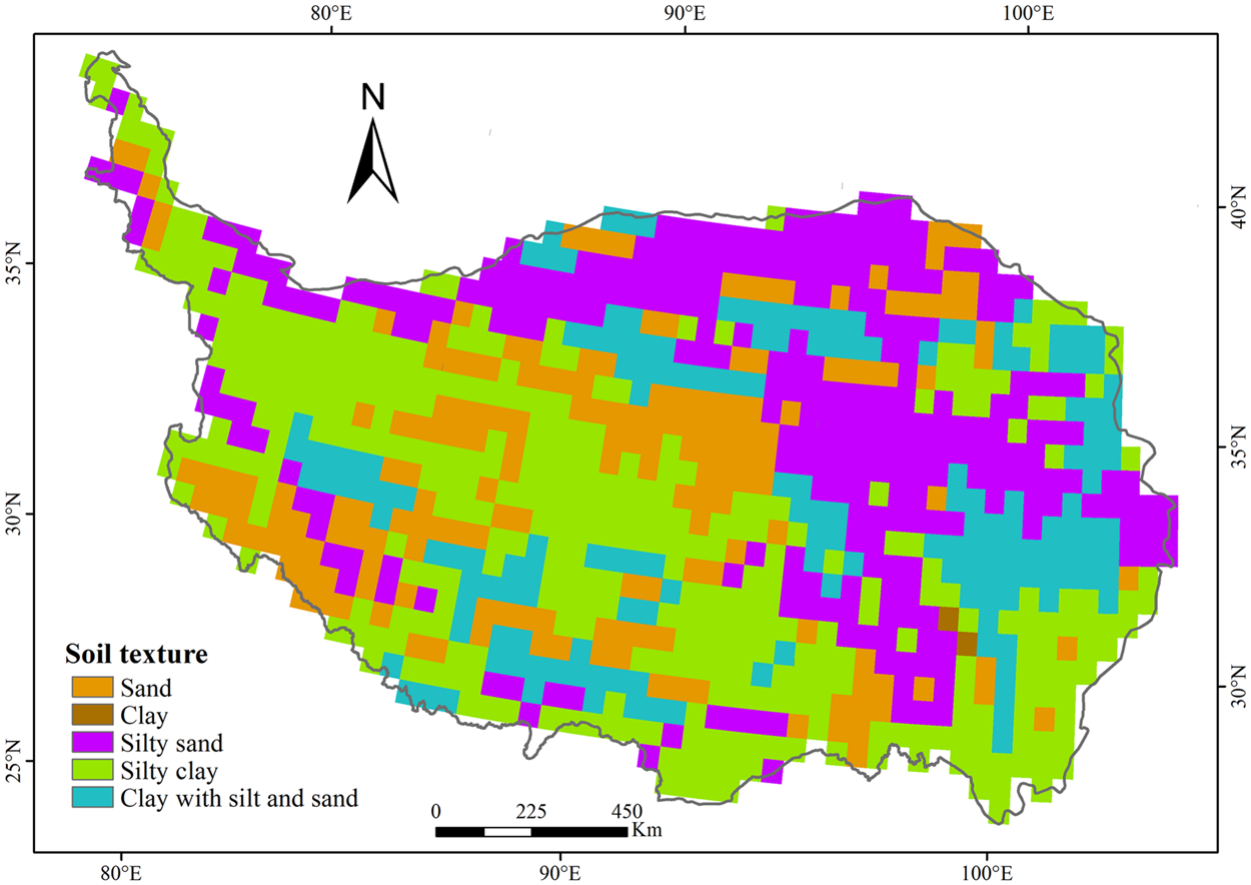
Spatial distribution of soil texture on the QTP. (The figure was created using ArcGIS 10.1 http://www.esri.com/ software).

Soil texture on the QTP was exhibited in Fig. 6. Soil texture from the map of soil texture types (1:14,000,000) was adopted in this study^79^, which embodies the information of the composition of soil particles. To match the resolution of climate data, soil texture data has been rasterized a raster with the resolution of 0.5° × 0.5°.
